# Evaluation of the Accuracy of Anthropometric Clinical Indicators of Visceral Fat in Adults and Elderly

**DOI:** 10.1371/journal.pone.0103499

**Published:** 2014-07-31

**Authors:** Anna Karla Carneiro Roriz, Luiz Carlos Santana Passos, Carolina Cunha de Oliveira, Michaela Eickemberg, Pricilla de Almeida Moreira, Lílian Ramos Sampaio

**Affiliations:** 1 Postgraduation Program of Medicine and Health, Federal University of Bahia, Salvador, Bahia, Brazil; 2 School of Nutrition, Nutrition Science Department, Federal University of Bahia, Salvador, Bahia, Brazil; 3 Faculty of Medicine, Department of Medicine, Federal University of Bahia, Salvador, Bahia, Brazil; 4 Nutrition Department, Federal University of Sergipe, Lagarto, Sergipe, Brazil; 5 Institute of Collective Health, Federal University of Bahia, Salvador, Bahia, Brazil; 6 Bahiana School of Medicine and Public Health, Salvador, Bahia, Brazil; 7 Postgraduation Program in Food and Nutrition, Federal University of Bahia, Salvador, Bahia, Brazil; Weill Cornell Medical College Qatar, Qatar

## Abstract

**Background:**

Visceral obesity is associated with higher occurrence of cardiovascular events. There are few studies about the accuracy of anthropometric clinical indicators, using Computed Tomography (CT) as the gold standard. We aimed to determine the accuracy of anthropometric clinical indicators for discrimination of visceral obesity.

**Methods:**

Cross-sectional study with 191 adults and elderly of both sexes. Variables: area of visceral adipose tissue (VAT) identified by CT, Waist-to-Height Ratio (WHtR), Conicity index (C index), Lipid Accumulation Product (LAP) and Visceral Adiposity Index (VAI). ROC analyzes.

**Results:**

There were a strong correlation between adiposity indicators and VAT area. Higher accuracy of C index and WHtR (AUC≥0.81) than the LAP and the VAI was observed. The higher AUC of LAP and VAI were observed among elderly with areas of 0.88 (CI: 0.766–0.944) and 0.83 (CI: 0.705–0.955) in men and 0.80 (CI: 0.672–0.930) and 0.71 (CI: 0.566–0.856) in women, respectively. The cutoffs of C index were 1.30 in elderly, in both sexes, with sensitivity ≥92%, the LAP ranged from 26.4 to 37.4 in men and from 40.6 to 44.0 in women and the VAI was 1.24 to 1.45 (sens≥76.9%) in men and 1.46 to 1.84 in women.

**Conclusion:**

Both the anthropometric indicators, C Index and WHtR, as well as LAP and VAI had high accuracy in visceral obesity discrimination. So, they are effective in cardiovascular risk assessment and in the follow-up for individual and collective clinical practice.

## Introduction

Visceral obesity is associated to higher incidence of type 2 diabetes, dyslipidemia, insulin resistance, hypertension, particularly cardiovascular disease (CVD) that are considered as important causes of mortality and high costs in the world [1], [2].

The quantification of visceral obesity is best determined by imaging exams such as Computed Tomography (CT), that is the gold standard method, but it requires high cost, difficult operation and radiation exposure. On the other hand, anthropometric clinical indicators are easily obtained and, if accurate, they offer diagnosis possibility in primary care and in the follow-up without any of the CT inconveniences. Currently few studies are available with data from adult and elderly subjects divided by sex to evaluate the accuracy of indicators in the prediction of visceral fat.

Recently, Lipid Accumulation Product (LAP) and Visceral Adiposity Index (VAI) have been proposed as alternative assessment parameters of the excessive lipids accumulation. The LAP expresses a continuous risk and it is a predictor of cardiovascular disease and mortality [3] and the VAI expresses the visceral fat function associated with cardiometabolic risk [4] and it also evaluates the risk of complications related to visceral obesity [4], [5]. Both have not been explored yet in regard to their ability to discriminate excess of visceral fat, measured by CT, as well as the Conicity Index (C Index) and Waist-to-Height Ratio (WHtR). In addition, there are few studies that evaluated the accuracy these indicators of visceral fat between adults and elderly individuals, in both sexes.

There are evidences that anthropometric indicators of abdominal obesity are good predictors of cardiovascular risk and mortality [6]–[10], but there are few studies that compare these indicators in relation to the LAP and the VAI as this study intended to do. The aim of this study was to determine the accuracy of anthropometric clinical indicators for discrimination of visceral obesity.

## Methods and Materials

### Study design and data collection

The transversal study was performed in the University Hospital and at the School of Nutrition of the Federal University of Bahia (UFBA), during the first trimester of 2009, conducted by the team of the Center of Studies and Intervention in Aging Area of UFBA in Salvador, the third largest city in Brazil. Two-hundred individuals from the outpatient clinic and the community were alocated by convenience for equitable stratification of variables: sex, age and body mass, the latter one determined by Body Mass Index (BMI), dividing patient weight in kilograms by the square of patient height in meters, specific for each age group.

The sample size was defined according to the possibilities of human and material resources, as well as the analysis of the sample size of previous studies [11]–[13] and the careful sample stratification. Individuals were selected following the same ratio between adults and elderly, men and women, presence and absence of excess body mass in order to achieve greater representativeness of the groups equally in terms of the amount of visceral fat, since the presence/absence of comorbidities has an influence in this amount of fat.

#### Exclusion criteria

individuals under twenty years old, Body Mass Index >40 kg/m^2^, patients with severe malnutrition and severe neurological and muscular disorders, pregnant and breastfeeding women, individuals that recently suffered abdominal surgery (<6 months) or had tumor, hepatomegaly, splenomegaly and/or ascites or any problem that compromises the recommended techniques for anthropometric and visceral fat measurement by computed tomography.

### Anthropometric clinical indicators

All clinical, anthropometric, laboratory and imaging by computed tomography evaluations were standardized and measured by a properly trained staff. Measurements of weight and height were obtained according to the techniques proposed by Lohman et al (1988) [14]. We directly measured weight in kilograms using a Portable, digital scale (Filizola, São Paulo, Brazil) with capacity up to 150 Kg and a precision of 100 g. The Height was measured in centimeters with a portable stadiometer (Seca, TBW Importing *Ltda.*). Waist Circumference (WC) was measured at the midpoint between the lower costal margin and the iliac crest, we used an inelastic measuring tape of 1 mm precision.

Measurements were obtained in duplicate and their averages were used for the analyzes. The WHtR was calculated by the WC (cm) divided by the height (cm). The Conicity index was obtained by the formula proposed by Valdez (1991) [15]:
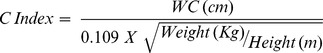



All individuals were submitted to a blood sample collection. They had fasted for 12 hours. The blood samples were used to measure the high-density lipoprotein (HDL) and triglycerides (TG) levels. They were quantified using a colorimetric system, dry chemistry method, with kits manufactured by Ortho-Clinical Diagnostics, Rochester, NY. For conversion between units, mg/dl to mmol/L, it was multiplied by 0.0113.

The VAI was obtained by the formulas proposed by Amato et al (2010) [4]: for males, VAI = (WC/36.58+(1.89×BMI))×(TG/0.81)×(1.52/HDL) and females, VAI = (WC/39.68+(1.88×BMI))×(TG/1.03)×(1.31/HDL). The LAP was obtained by the formulas proposed by Kahn et al (2005) [3]. For males, LAP = (WC [cm]–65)×(triglyceride concentration [mmol/L]) and females, LAP = (WC [cm]–58)×/triglyceride concentration [mmol/L]).

### Tomographic image of Visceral Adipose Tissue area (VAT)

The area of Visceral Adipose Tissue was measured by computed tomography obtained by the CT Spirit Siemens of the Radiology Service from the University Hospital of UFBA and analyzed by a single specialized technician. The examination was performed in complete fast of 4 hours without administration of barium or organic iodinated contrast, with the patient in supine position and the arms extended overhead. The area was obtained from a single axial CT slice at the level of L4–L5, with a slice thickness of 10 mm and an exposure time of three seconds, according to a technique proposed by Seidell et al (1987) [16]. The VAT area was described in square centimeters.

The tomographic program was used with x-ray CT scanner parameters of 140 kV and 45 mA, being employed the density of −50 and −150 Hounsfield units for the identification of fat tissue. Visceral obesity was identified by the VAT area above 130 cm^2^ [17].

### Statistical Analyzes

For descriptive analysis it was used the means, standard deviations, as well as the Kolmogorov-Smirnov test and the histogram to assess the distribution of the variables. In adittion, we used Student t-test and the Wilcoxon test to compare the mean of the variables of normal and non-normal distribution, respectively. The coefficient of variation was calculated to assess the inter and intra examiner variability of the anthropometric measures. The correlations by the Pearson’s and Spearman’s correlation coefficient according to the variables linearity.

The areas under the ROC curves (Receiver Operating Characteristic) (AUC) were calculated by adiposity indicators in the identification of discriminatory power for visceral obesity. The area under ROC curve value equal 1 means perfect accuracy of diagnosis and the closer to this value and greater than 0.75, it is, it has a precision sensibility [18]. Sensibility, specificity, positive predictive value (PPV) and negative predictive value (NPV), and their respective cutoffs with more appropriate balance between them were examined. A confidence interval (CI) of 95% was adopted. The significance level was set at p<0.05. For the analyzes it was used the statistical program SPSS; version 16.0 (SPSS Inc., Chicago, IL, EUA).

### Ethics Statement

This study was approved by the Ethics Committee in Research of the School of Nutrition (license numbers: 01/09), Federal University of Bahia, in Salvador, Brazil. All participants agreed in participating in this research by signing a written informed consent. The study did not involve procedures of high risk for the individuals and all received the test results, and they were seen at nutrition clinics of the University Hospital and referred for health follow-up, where necessary.

## Results

Two-hundred individuals were evaluated, from whom 9 were excluded (TG values >400 mg/dl and outliers of the visceral fat), totaling 191 participants in this study. The anthropometric data obtained correlation coefficient of intra and inter evaluator exceeding 0.90 confirming the reliability of the measures collection. In Table 1, the mean WHtR was 0.57 and 0.59 in elderly men and elderly women, respectively. The mean C Index was higher in the elderly group of both sexes. It was verified that the LAP and VAI had higher means in elderly comparing with group of adults, in both sexes, however this difference did not have statistical significant, except the mean of the LAP in women. The average area of VAT was over 130 cm^2^ only in elderly men (p<0.05) (Table 1).

**Table 1:** Descriptive analysis of the anthropometric clinical indicators and visceral adipose tissue area, and the mean comparison of these variables, by sex and age group.

	Men			Women		
	20–59 years (n = 49)	60 years (n = 45)	p	20–59 years (n = 49)	60 years (n = 45)	p
**WHtR**	0.51 (0.07)	0.57 (0.07)	0.001**	0.53 (0.07)	0.59 (0.07)	0.001**
**C Index**	1.23 (0.09)	1.31 (0.08)	0.001**	1.21 (0.08)	1.29 (0.09)	0.001**
**VAI**	1.57 (0.98)	1.73 (1.08)	0.470	1.67 (1.33)	2.15 (1.44)	0.090
**LAP**	35.66 (31.07)	43.27 (30.64)	0.240	34.12 (27.66)	47.60 (26.98)	0.020*
**VAT**	94.18 (58.74)	157.80 (86.08)	0.001**	72.20 (43.88)	122.53 (48.94)	0.001**

Data are mean_S.D.

Abbreviations: WHtR: Waist-to-height ratio; C Index: Conicity Index; VAI: Visceral adipose index; LAP: Lipid accumulation product; VAT: Visceral adipose tissue area (cm^2^).

*p≤0.05;

**p≤0.01.

In both sexes it was found that anthropometric indicators had strong correlations with the VAT area, increasing from the group of adults to the one of older men and in women the inverse was observed. The LAP showed strong correlation with VAT area, r≥0.70 (p≤0.01) and r≥0.60 (p≤0.01) respectively in men and women. The strongest correlation of the VAI was r = 0.56 (p≤0.01) in elderly men, while in elderly women the correlation was moderate (Table 2).

**Table 2:** Correlation coefficient between visceral adipose tissue area and the indicators of adiposity in both sexes according to age group.

	Visceral Adipose Tissue Area			
	Men		Women	
	20–59 years	≥60 years	20–59 years	≥60 years
**WHtR**	0.79**	0.80**	0.73**	0.64**
**C Index**	0.68**	0.82^1^ **	0.72**	0.47**
**VAI**	0.50^1^ **	0.56^1^ **	0.38**	0.47**
**LAP**	0.70^1^ **	0.73^1^ **	0.61**	0.60**

Abbreviations: WHtR: Waist-to-height ratio; C Index: Conicity Index; VAI: Visceral adipose index; LAP: Lipid accumulation product.

**p≤0.01.

^1^Spearman’s correlation coefficient.

**Figure 1:**
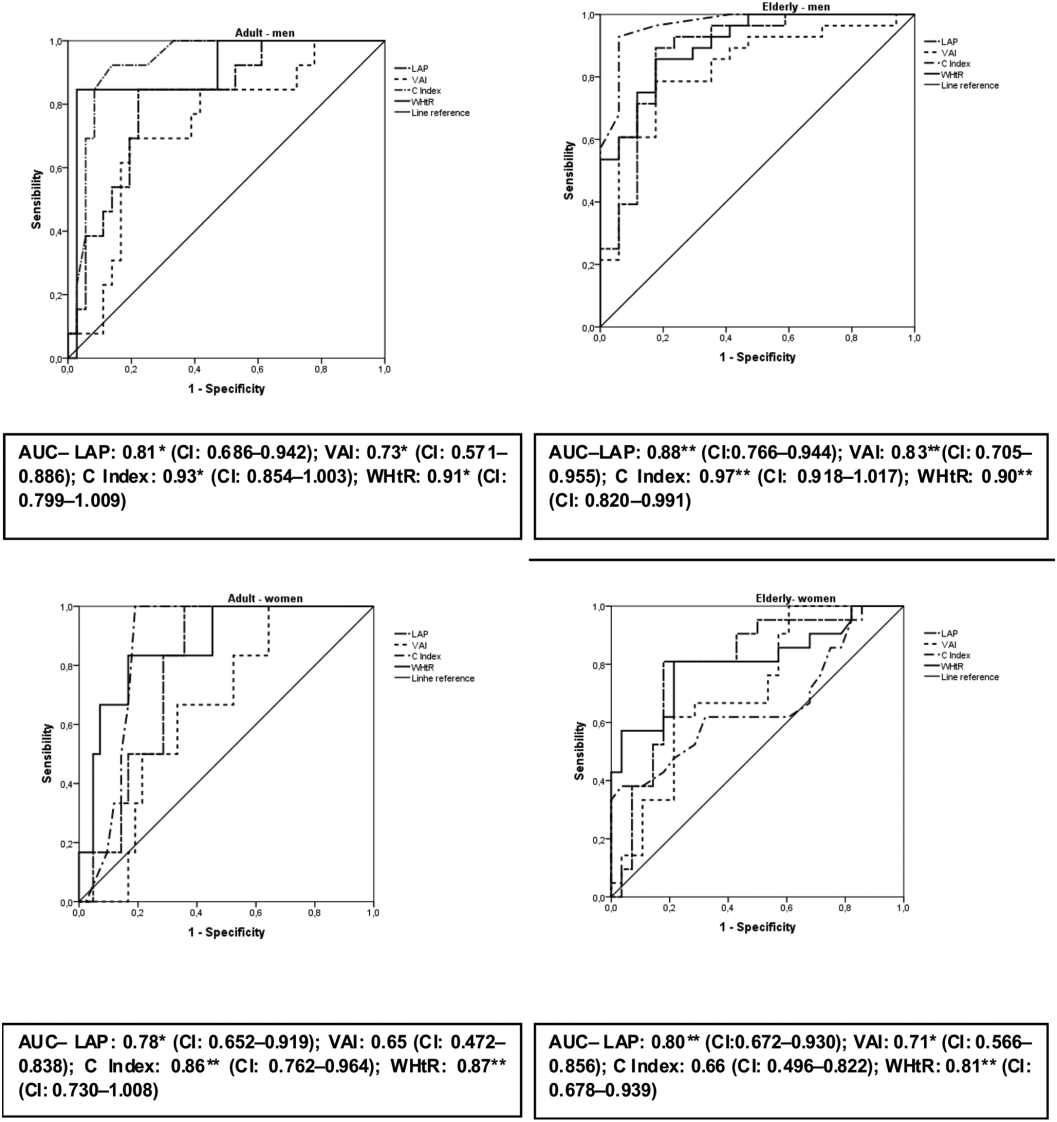
ROC analysis with Areas Under the Curve (AUC) of indicators of adiposity in predicting visceral obesity (TAV area >130 cm^2^). In this Figure are presented the areas under the ROC curves of anthropometric clinical indicators, classified by sex and age groups. In both sexes the majority of indicators had strong predictive ability to detect the presence of visceral obesity, i.e., AUC above 0.80. Abbreviations: AUC: Areas under the ROC curves, CI: Confidence Interval; LAP: Lipid accumulation product; VAI: Visceral adipose index; C Index: Conicity Index; WHtR: Waist-to-height ratio. *p≤0.05; **p≤0.01.

In Figure 1 and Table 3, the analyzes of the ROC curves of anthropometric clinical indicators are presented. In both sexes the WHtR and C Index showed a higher area under the ROC curve than the LAP and the VAI, i.e., AUC≥0.90 in men and AUC≥0.86 in women, except in elder women. The VAI had larger areas in elderly, with AUC = 0.83 (CI: 0.705–0.955) in men and AUC = 0.71 (CI: 0.566–0.856) in women. Regarding other indicators, the VAI had the lower discriminatory power (Figure 1).

**Table 3:** ROC analysis with Cutoff points, sensitivity and specificity of indicators of adiposity that correspond to VAT area of ≥130 cm^2^ for men and women according to age group.

	20–59 years			≥60 years		
	Cutoff	Sens. (PPV)	Spec. (NPV)	Cutoff	Sens. (PPV)	Spec. (NPV)
**MEN**						
**WHtR**	0.54	84.6 (68.7)	86.1 (93.9)	0.55	85.7 (88.9)	82.4 (77.8)
**C Index**	1.26	92.3 (70.5)	86.1 (96.9)	1.30	92.9 (91.6)	94.1 (89.0)
**VAI**	1.45	76.9 (41.6)	61.1 (88.0)	1.24	78.6 (84.6)	76.5 (68.5)
**LAP**	37.4	84.6 (57.9)	77.8 (93.3)	26.4	92.9 (94.7)	76.5 (86.7)
**WOMEN**						
**WHtR**	0.59	83.3 (41.6)	83.3 (97.2)	0.58	81.0 (74.0)	78.6 (84.6)
**C Index**	1.25	100 (42.9)	81.0 (100)	1.30	61.9 (59.2)	67.9 (70.3)
**VAI**	1.46	66.7 (22.2)	66.7 (93.3)	1.84	66.7 (60.9)	67.9 (73.1)
**LAP**	40.6	83.3 (29.4)	71.4 (96.8)	44.0	81.0 (77.3)	82.1 (85.2)

Abbreviations: Sens: Sensitivity; Spec: Specificity; PPV: Positive predictive value; NPV: Negative predictive value; WHtR: Waist-to-height ratio; CI: Conicity Index; VAI: Visceral adipose index; LAP: Lipid accumulation product.

It is observed in Table 3 that the cutoffs of WHtR were similar between age groups, in both sexes, with sensibility and specificity above 81.0% and 78.6%, respectively. Regarding C Index, elderly had cutoffs of 1.30, with sensibility and specificity of 92.9% and 94.1% in men and 61.9% and 67.9% in women, respectively (Table 3).

The LAP cutoffs ranged from 26.4 to 37.4 in men, reaching a PPV of 94.7% with sensibility and specificity of 92.9% and 76.5% respectively, in the men older than sixty years while in women the LAP ranged from 40.6 to 44.0, with sensibility and specificity of 81.0% and 82.1%, respectively in elderly women. The VAI cutoffs were 1.45 (sens: 76.9%; spec: 61.1%) and 1.24 (sens: 78.6%; spec: 76.5%) in adults and elderly men, respectively. Among women the cutoffs were 1.46 in adults and 1.84 in elderly with sensitivity and specificity above 66.7% (Table 3).

## Discussion

The present study showed that the clinical anthropometric indicators had strong correlations with visceral fat and most of the areas under the ROC curve gained predictive power with the increasing of age, being more expressive, especially in men. This is the first study that evaluates the accuracy of the LAP and the VAI compared to anthropometric indicators for the detection of visceral obesity as measured by computed tomography in both sexes.

In this study, the WHtR and C Index evaluated well the visceral obesity. The C Index incorporates three very instructive measures, including the WC, which is common to the other indicators here evaluated. The C Index proved to be one of the most accurate in the discrimination of the visceral obesity, especially in men. It detects the changes in fat distribution, allowing comparisons between individuals that had different measurements of body weight and height [19]. Studies show that C Index has been a good predictor of high coronary risk [20] with AUC of 0.80 (95% CI: 0.74–0.85) and cardiovascular risk [21], as well as the WHtR [22].

The WHtR allows identifying the waist circumference of the risk to an individual’s height and was better than the C Index only in the older women in this study. A systematic review showed that the WHtR was better than WC and BMI in predicting CVD in 86% of studies in men and in 91% in women [23].

Regarding the LAP, there was an increase of accuracy comparing younger and elderly individuals in both sexes in this study. The LAP estimates the over-accumulation of lipids, rising more quickly with the age for men than for women, which may contribute to the increasing in cardiovascular events in younger men [3]. In this study, the LAP and the WHtR had similar accuracy (AUC = 0.80) to identify visceral obesity in older women. Studies showed the discriminatory power of the LAP for other events, such as the incidence of diabetes that was similar to waist to hip ratio and WHtR [24] and in the prediction of metabolic syndrome where there was superiority of the LAP in relation to WHtR [25]–[27].

In this study, despite the VAI having positive correlation with visceral fat, it had shown lower accuracy compared to other indicators, in both sexes, however there was good accuracy in the elderly. In the same way as the LAP, the VAI has been reported to predict other events resulting from excess of visceral fat [28]–[30] not of visceral obesity itself, as in the present study.

Overall, the most indicators of this study demonstrated that elderly men had better correlation and accuracy in discrimination of the excess of visceral fat than adults men. The inverse was observed to women, possibly because of the physical and hormonal changes that happen to women in the postmenopausal. However, this more detailed investigation wasn’t the goal of this study. Furthermore, the LAP and the VAI showed lower accuracy comparing to the WHtR and the C Index because of bioquimics tests (TG, HDL), which constitute their respective equations and have weaker correlations with visceral fat than the anthropometry [31].

The cutoff points of the indicators evaluated in this study to identify the visceral obesity are still a gap in the literature. The values for the elderly, who have not been previously identified are of particular interest. For the WHtR, the cutoffs were similar to those found in other studies to identify high coronary risk [32], [33] and to discriminate diabetes, hypertension and dyslipidemia [34]. However, we didn’t find other studies about the discrimination of visceral obesity, which showed the cutoff of anthropometric clinical indicators, as this study does. Therefore, it is recommended to consider that the waist should not be greater than half of the height of a particular individual as this would indicate a health risk.

The theoretical range of the C Index is between 1.00 and 1.73 and this ratio increases with the accumulation of abdominal fat, already taking into account its height and weight, increasing the risk of diseases [35]. In this study the C Index was able to detect more cases of visceral obesity in men than in women, with values from 1.26 to 1.30 in adults and elderly respectively. Studies have suggested cutoffs of the C Index in order to identify high coronary risk and hypertension [9], [36], however, they were assessed only in adults and not in the elderly.

For the LAP, this study identified values above 26.4 and 40.6 in men and women respectively, i.e., values for women were superior than for men to detect visceral obesity. There is information about the LAP cutoffs to detect other events, among them, a study with spanish adults [26] identified the LAP value above 48.9 in men and 31.7 in women to detect metabolic syndrome. In relation to the VAI, cutoffs were similar among adults, while among the elderly, men had a value of 1.24, being lower than that of women that was 1.84. The results presented here show that values above those cutoffs allow to estimate visceral obesity and underscore the importance of obtaining and using specific cutoffs points for each population.

The scarcity of studies about this subject and which evaluated anthropometry with the LAP and the VAI, using this imaging method restricted the comparison of results between the tested age groups. As shown in most studies [2], the measurement of single computed tomography scan, especially at L4–L5 position minimizes radiation exposure of the individual and reduces cost. This way we have chosen this position this study. Further studies are needed, with larger samples, allowing generalizations and comparisons among different populations.

This study reinforces that individuals should be assessed early and periodically, through accurate methods, considering its ease of use in large scale and low cost, especially those one that replace the CT, such as WHtR, C Index, LAP and VAI, enabling a better nutritional clinical evaluation in elderly and adults able to intervene effectively for the prevention and/or treatment of visceral obesity related to cardiovascular risk.

In conclusion, visceral obesity, considered a large spectrum of cardiovascular risk, was highly discriminated by both anthropometric indicators, C Index and WHtR, as well as by the LAP and the VAI. Therefore, these methods are effective tools in cardiovascular risk assessment and in the follow-up for individual and collective clinical practice.
