# Correction: Evaluation of the Accuracy of Anthropometric Clinical Indicators of Visceral Fat in Adults and Elderly

**DOI:** 10.1371/journal.pone.0116449

**Published:** 2014-12-19

**Authors:** The PLOS ONE Staff

There are errors in the first sentence of the fourth paragraph of the “Anthropometric clinical indicators” subsection of the Methods and Materials. The correct sentence is: The VAI was obtained by the formulas proposed by Amato et al. (2010) [4]: for females, VAI =  (WC/36.58 + (1.89 x BMI)) x (TG/0.81) x (1.52/HDL) and males, VAI =  (WC/39.68+ (1.88xBMI)) x (TG/1.03) x (1.31/HDL).
